# Cognitive effectiveness of high-intensity interval training for individuals with methamphetamine dependence: a study protocol for randomised controlled trial

**DOI:** 10.1186/s13063-021-05615-9

**Published:** 2021-09-23

**Authors:** Shen Menglu, Yang Suyong, Wang Xiaoyan, Wolfgang I. Schöllhorn, Zhu Dong

**Affiliations:** 1 Wushu College, Shanghai University of Sport, 399 Changhai Road, Shanghai, China; 2 School of Sport Psychology, Shanghai University of Sport, Shanghai, China; 3 School of Physical Education, Hangzhou Normal University, Zhejiang, China; 4 Institute for Sport Science, Johannes Gutenberg-University Mainz, Mainz, Germany; 5 School of International Education, Shanghai University of Sport, 399 Changhai Road, Shanghai, China

**Keywords:** Methamphetamine, High-intensity interval training, Moderate-intensity continuous training, Cognitive function, Inhibitory control

## Abstract

**Introduction:**

Cognitive deficit is a common syndrome of methamphetamine (MA) dependence. It is related to decision-making, control ability, and social functioning. High-intensity interval training (HIIT) is a training technique that requires people to work out at full intensity during a short period. Many studies have already shown the potential effects of HIIT on cognitive functions. The purpose of this trial is to evaluate the cognitive effects of HIIT on individuals with MA dependence.

**Methods and analysis:**

A total of 240 individuals with MA dependence will be randomly assigned to the HIIT group, moderate-intensity continuous training (MICT) group and control (CON) group. HIIT will consist of a 24-min HIIT exercise on a treadmill. MICT will consist of a 1-h body–mind exercise. CON will be their traditional intervention. The experimental period will be 12 months with 3 interventions weekly for the first 6 months and follow-up for the next 6 months. All subjects will be given cognitive tests at baseline, after intervention and at follow-up. Cognitive performances will be compared by a mixed-model analysis for repeated measures.

**Discussion:**

HIIT training may reduce illicit drug cravings amongst individuals with MA dependence; hence, HIIT may have a good effect on the cognitive functions, such as memory and executive function, of individuals with MA dependence.

**Trial registration:**

Chinese Clinical Trial Registry ChiCTR2000032492. Registered on April 30, 2020 (http://www.chictr.org.cn/edit.aspx?pid=52127&htm=4)

## Introduction

Substance abuse is a growing public health concern worldwide that causes negative influences to the health of abusers and leads to the occurrence of illegal and criminal behaviours. Methamphetamine (MA) consumption has risen rapidly in the past few years with an estimated annual prevalence of 0.7% worldwide [85]. By the end of 2019, 2.1 million drug dependents in China have been recorded; amongst whom, 1.2 million were individuals with MA dependence [71]. Compared with traditional illicit drugs, such as opium and heroin, MA causes greater harm to the human body [32]. Besides arousing users’ strong mental dependence, MA also directly affects the central nervous system and causes irreversible damage [58, 98]. Numerous long-term MA users suffer consequences, such as physical illness, mental health, social adjustment and poor mental symptoms [48, 80]. These consequences may be because MA disrupts the prefrontal striatum dopamine pathway involved in various cognitive and psychomotor processes, including behavioural decision-making [55]. Long-term MA use can lead to a series of cognitive deficits [12, 96]. Amongst which, the most serious are related to reward or impulsiveness and social cognition [73]. Such deficits are associated with decision-making disorders caused by drug addiction [70]. Unlike normal subjects, those who have used MA for more than 5 years have a defect in decision-making ability in balancing reward and punishment [55]. MA use changes the decision-making ability by altering the value perception of reward and negative results [68], which could lead addicts to continue to make negative decisions despite the potential hazard of the reward stimulus [95]. Thus, drug users tend to lose control of their drug-taking and drug-seeking behaviours [69]. Data from several Stroop tests, including alcohol, cocaine, nicotine and internet addiction, pointed out that compared with the normal control group or patients with light symptoms, addicts have poorer concentration, reaction time and accuracy under addiction-related stimulus (such as drug pictures), which represent their poor inhibitory control [26, 31, 35, 45, 61]. Similarly, another research showed that individuals with MA dependence have delayed cognition, misjudgement and poor cognitive control in the Stroop test using MA-related words as irrelevant stimuli [20]. Another drawback of long-term MA use is attention deficit [7]. MA and cocaine dependents show worse attention in the Stroop test than healthy control subjects [50, 79]. In summary, individuals who are chronically exposed to illicit drugs experience difficulties in execution, inhibition and decision-making [7].

Exercise as a supplementary treatment for substance abuse has been favoured and concerned by many countries [2]. Exercise can reduce withdrawal symptoms and the possibility of relapse [13, 83]. Theoretical and empirical studies have found that short- and long-term moderate-intensity aerobic and resistance exercise may remarkably improve the brain cognition, psychological behaviour and physical function of the drug-dependent group, as well as ameliorate their life quality and drug craving [62, 89]. One study found that the mental health of heroin dependents is promoted after 6 months of aerobic exercise; the effect of aerobic exercise intervention appears at 3 months, whereas yoga exercise needs 6 months to take effect [81]. Another study [90] manifested lower levels of craving and greater accuracy in behavioural inhibition control in individuals with MA dependence who underwent aerobic exercise for 12 weeks. Cardiovascular function and physical condition are remarkably improved, the nervous system tends to balance and the inhibitory ability of MA and drug craving are ameliorated in amphetamine-type synthetic drug dependents after 6 months of tai chi (TC) intervention [49, 66, 97].

High-intensity interval training (HIIT) is a relatively short repetitive training performed in an ‘all-out’ manner at an intensity that can cause VO_2_ peak and usually lasts from 10 s to 4 min with short intervals [15, 40]. HIIT is able to achieve a certain physical activity level in a shorter time than traditional aerobic exercise or physical activity. HIIT is a recently popular form of full-body aerobic exercise that includes various exercises, such as running and roping. Aerobic exercise has a preferable influence on cognitive function, including memory and reaction time [6, 60], and the effect of HIIT on physical and mental health is more helpful than moderate-intensity continuous training (MICT). Besides, exercises of different intensities have disparate effects on cognitive function; a study showed that cognitive function is enhanced immediately after a high-intensity workout [6]. A meta-analysis also found that executive effects, including inhibitory control and memory task, tend to improve during a series of cognitive tasks within a period of time after acute high-intensity exercise [18]. Additionally, the positive effect of acute high-intensity exercise on cognitive performance is more persistent than that of MICT [18]. Some studies suggest a positive correlation between exercise intensity and cognitive function [30, 34, 54]. Moreover, the effects of aerobic exercise on cognition depend on exercise intensity [36]. Notably, as an important cognitive mechanism, increasing research has linked inhibitory control with higher cognitive functions [41, 91]. Poor inhibition control is likely an element in the maintenance of addiction [87] and a key factor in the prediction of the successful recovery of addicts [9, 32, 35, 38]. Physical activity has a host of potential advantages in addiction treatment [2, 93]. However, drug addicts have difficulties in adhering to a certain type of exercise for long periods of time because of comorbid cognitive and mental health problems [2]. Together, these studies indicate that the results of exercise intervention will be affected, and HIIT can solve this issue to some extent owing to its less time consumption.

Investigations on MICT and HIIT to improve cognitive function have recently gained much attention. Although a few studies explored the effects of HIIT on drug-dependent animals [47], research on the cognitive effects of HIIT on drug dependents, especially MA dependents, is lacking. The aims of this trial are to (1) determine whether HIIT improves the cognitive function of individuals with MA dependence, (2) determine whether HIIT reduces drug craving and improves the mental health of individuals with MA dependence and (3) compare the cognitive rehabilitation effects of HIIT and MICT on individuals with MA dependence. We hypothesise that (1) HIIT and MICT interventions will be effective in improving the cognitive functions of MA dependents compared with the control group and (2) the effect of HIIT will be better than the other two interventions (i.e. MICT and control).

## Methods

### Study design

**Fig. 1:**
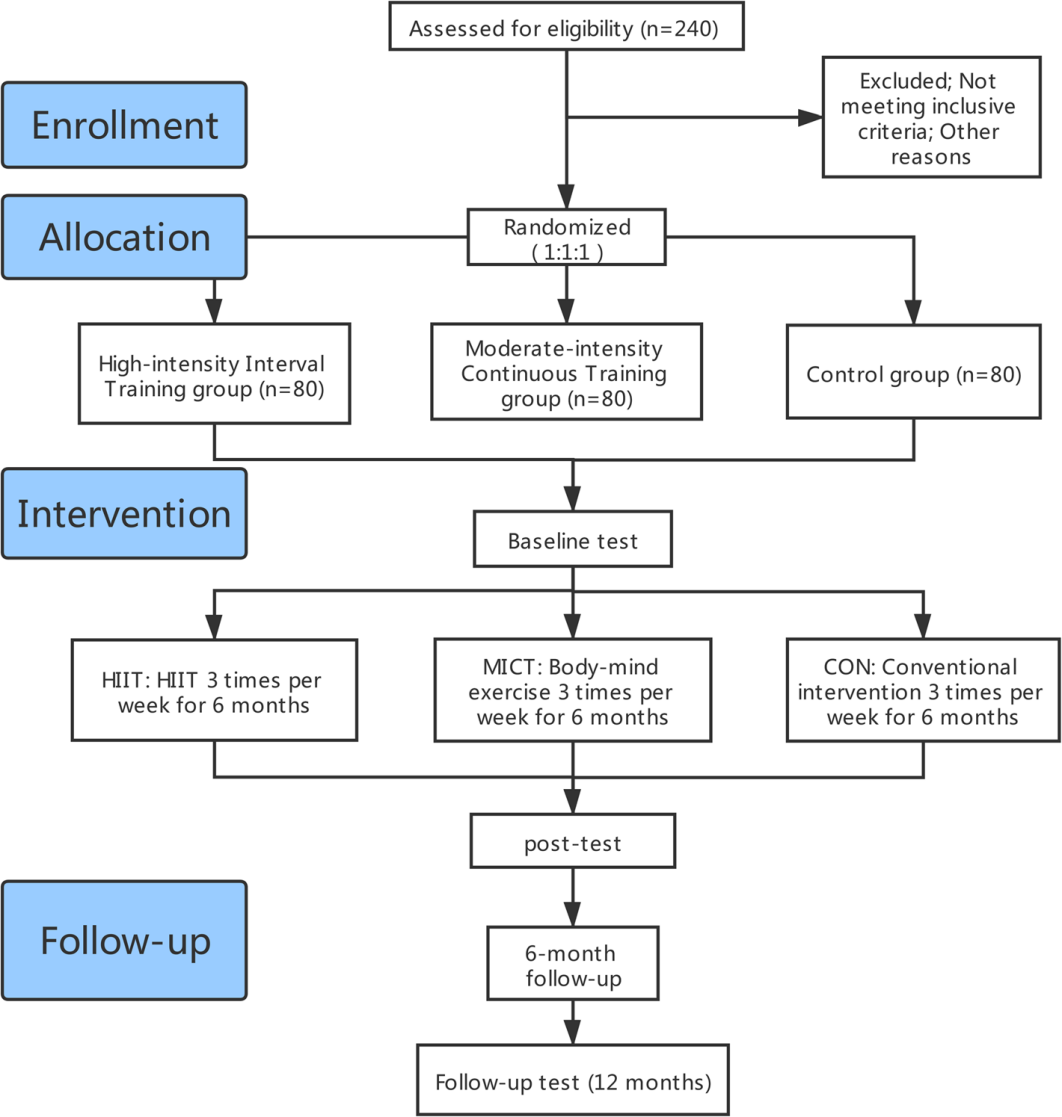
Flow diagram of the study design

This study is designed as a prospective, single-blind randomised controlled trial (RCT). The overview of the trial is displayed in Fig. 1.

### Design and procedures

**Fig. 2:**
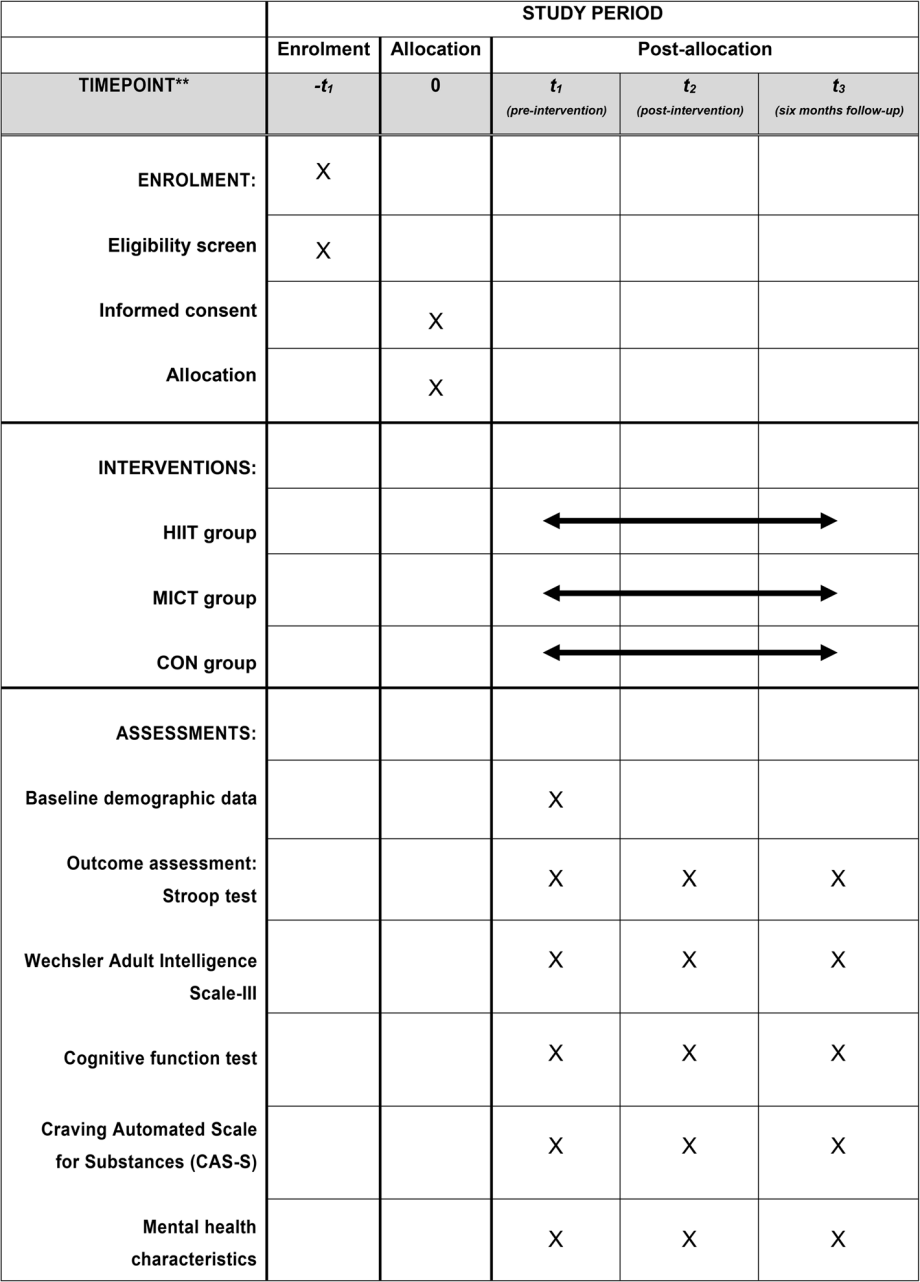
Schedule of enrolment, interventions and assessments

We will publish study posters in Shanghai Mandatory Detoxification and Rehabilitation Centre (SMDRC) to recruit individuals with MA dependence. Subjects who volunteer to participate in and complete the whole experiment will be rewarded additional number of phone calls with family members. Enrolled participants will be randomly assigned to three groups. Participants in the HIIT group will run 24 min on a treadmill in the HIIT programme; participants in the MICT group will practise body–mind exercise in a fixed, quiet site, and participants in the control group will maintain their ordinary daily intervention, including health knowledge education and recreational aerobics exercise. The experimental groups will receive professional guidance, and the control group will undergo intervention under the guidance of SMDRC. The total experimental period will be 12 months. The first 6 months will entail exercising intervention three times a week for a total of 72 times. All the interventions will be conducted simultaneously. Subjects who will complete 80% of the total intervention times are eligible to participate in the subsequent evaluation. The three groups will be given cognitive tests at baseline, after the intervention and at the 6th month of follow-up (Fig. 2). In the 6-month follow-up observation, all subjects will continue their previous normal rehabilitation course in SMDRC as required before under the supervision of their previous administrators. All subjects selected will voluntarily participate in this experiment and sign the informed consent prior to the study. Additionally, the auditing trial conduct procedure will be independent from the patients and the sponsor.

### Participants

#### Inclusion criteria

The patients must meet the following criteria for inclusion in the study: (1) age between 18 and 40 years, (2) drug dependence diagnosed according to the Chinese Classification of Mental Disorders 3, (3) remaining withdrawal time of more than 1 year, (4) absence of serious medical or mental illness, (5) primary school education or higher, (6) compulsory drug treatment and (7) volunteered to participate in this study.

#### Exclusion criteria

The patients will be excluded if they have any of the following: (1) participation in HIIT before the present study, (2) other diseases that may interfere with the implementation of the research programme, such as heart, lung, liver and kidney diseases, musculoskeletal, dysfunction or cancer, (3) antisocial personality disorder and borderline personality disorder, (4) severe cognitive, visual or auditory impairment and (5) unstable medication.

#### Dropout and suspension criteria

Subjects will have the right to withdraw from the study at any time during the intervention period. When they feel uncomfortable during the intervention, they can withdraw from the present study according to medical advice. Additionally, the subject will be excluded if an unexpected worsening of motor or neuropsychiatric symptoms occur.

### Randomisation and blinding

The study was designed as a single-blinded RCT. Participants will be randomly assigned into three groups by two trained assessors who will not participate in the intervention. A sequence number will be generated by SPSS 25.0 under the ratio of 1:1:1. The numbers will be placed in a sealed envelope and randomly distributed to the participants. The instructors in each group will be blinded to the group allocation, including the control group, and the instructors should not know each other. This study will be unable to blind participants because of the differences in the intervention methods to be used in the three groups.

### Sample size calculation

Sample size estimation was conducted using G*Power V.3.1 (Franz Faul, Universitat Kiel, Germany). The purpose of this study is to examine the effects of HIIT on the major cognitive functions of MA dependents. However, informed preliminary data and empirical evidence on the effects of HIIT are lacking. Thus, we estimated the effect size in these power calculations using an estimated sample from published studies that compared the effects of HIIT and continuous training on cognitive function in healthy adults [65]. Specifically, we used the values of some variables within the ANOVA framework in this essay to obtain an effect size of 0.28 through the *t* test. Then, according to the *F*-test at *α* = 0.05 and power = 0.95, we obtained the required sample size of 223 subjects. The final average enrolment number with an anticipated 10% attrition rate is 240 (80 in each group) for the study to detect the difference in the cognitive outcomes between HIIT and MICT relative to the control group.

### Intervention

#### HIIT group

The HIIT group will undergo three interventions per week for 6 whole months on a treadmill. Total exercise time is 24 min, including 3 min of warm-up with low intensity (walking or jogging), 18 min of high-intensity training and moderate-intensity intervals and 3 min of relaxation (walking or jogging). The 18-min HIIT process will consist of six groups of 2-min high-intensity exercises and 1-min moderate-intensity intervals. Training will be conducted under the supervision of professional instructors. During the supervised intervention, heart rate (HR) will be recorded using a Polar HR monitor (model FT4; Polar Electro, Finland) to encourage or ensure that an appropriate exercise intensity is maintained according to their measured maximum HR. In addition, each subject will be able to observe their exercise intensity and real-time HR from a 6 × 3 m^2^ projector curtain.

Different parts of the intervention will have different exercise intensities. Warm-up will be 50–65% of the maximum HR, and the 2-min high-intensity training will be 80–90%. The 1-min moderate intermittent exercise will be 70–80% of the maximum HR. The target intensity of relaxation will be 50–65% of the maximum HR in theory [75]. Exercise intensity will be controlled by regulating the speed of the treadmill. All interventions will be implemented in the afternoon. Except for the different intervention parts, the participants were advised to carry out their daily rehabilitation staying the same carrying out by the administrators and will be instructed to remain their usual lifestyle (such as diet and medication).

#### MICT group

The MICT protocol will consist of TC rehabilitation exercises and self-designed body–mind exercises. Twenty-four-style TC movements, including ‘part the wild horse’s mane on both sides, hold the lute, forearm on both sides, grasp the bird’s tail, brush knee and twist step on both sides and golden rooster standing on one leg’ will be reserved in the TC rehabilitation exercise, and the entire exercise duration will be nearly 4–5 min. The self-designed body–mind intervention will consist of nine forms of exercise, which are tailored to the physical and mental characteristics of illicit drug users. These movements combine TC, Qigong and Yoga and emphasise dynamic postural control and body weight shift stepping with lateral–medial and anterior–posterior movements, body symmetry pulling across up–down and left–right axes and hand-eye coordination movement. The duration of each session is 1 h. The frequency of intervention in the MICT group will be the same as those of the HIIT and control groups. Exercise intensity will be achieved by adjusting the movement range and squat height under the guidance of professional instructors.

#### Control group

The control group will be instructed to continue their normal intervention, including relevant health education, as well as their daily activities, such as reading books and watching news. The intervention in this group will be conducted under the supervision of an administrator from the rehabilitation centre. The subjects and the administrator should not know each other.

### Incremental exercise test

The primary purpose of this test is to obtain the maximum HR of the HIIT group to minimise or avoid the risk that may occur during the formal intervention. Participants will be tested on a power bike following the cardiopulmonary test guidelines of the American Heart Association [8] after knowing the procedure of the experiment. Borg 6–20 scale will be used to measure motion perception every 2 min [14]. The test will stop when the subjects reach the maximum voluntary fatigue index of 18–20 as determined by the scale, that is, when the participants are unable to reach the established rhythm (< 5 RPM) for 5 s or they show signs of extreme fatigue.

### Adverse events

The safety of patients will be monitored during the intervention and their daily life. Participants will be informed to contact the administrators in the rehabilitation centre in case of any adverse event situation. In addition, participants will be able to discontinue the study once they experience any symptoms of discomfort or adverse event.

### Study assessments

#### Participants’ characteristics

The demographic and health characteristics of subjects will be collected at baseline (Table 1). The characteristics will include age, gender, education, marital status, drug abuse years, type of drug use, whether exercise, weight (kg), height (cm), body mass index, blood pressure (mmHg) and lung capacity (ml). Automatic equipment (Omron sphygmomanometer), electronic digital scales and electronic lung capacity tester will be used to measure blood pressure, weight, height and lung capacity, respectively.

**Table 1:** Demographic and characteristics of participants

	HIIT (*n=80*)	MICT (*n=80*)	CON (*n=80*)
Age			
Gender			
Education			
Marital status			
Drug abuse years			
Types of drug use			
Whether exercise			
Weight (kg)			
Height (cm)			
BMI			
Hypertension (mmHg)			
Pulmonary (ml)			

### Primary outcome assessment

#### Stroop test

Stroop is one of the most widely used neurocognitive tasks [63, 67] and as a tool to assess the efficiency of inhibition mechanisms and explore the relationship between inhibition and impulsivity [72]. Individuals with substance use disorders have lower baseline metabolic activity in the prefrontal cortex (PFC), which is associated with impaired cognitive function in decision-making and inhibitory control [4, 11]. The cognitive portion of the Stroop test has been used as a psychometric test to assess cognition as it is related to executive function in decision-making and inhibitory control related to PFC exercise.

The experimental materials are ‘RED’, ‘GREEN’ and ‘BLUE’ characters written in Chinese, and conditions will be classified as congruent and incongruent (Table 2). Congruent condition means that the font colour is in line with its literal meaning, whereas incongruent condition means that the font colour disagree with its literal meaning. Participants will be asked to respond to the colour of the word through the following instructions: press ‘J’ key if the colour of the Chinese character is red, press ‘K’ if it is s green and press ‘L’ if it is blue. Keys ‘J’, ‘K’ and ‘L’ correspond to the index finger, middle finger and ring finger of the right hand, respectively. At the beginning of the experiment, the fixation point ‘+’ will be presented in the centre of the screen for 500 ms, and then the experimental stimulus is presented. Participants will be required to identify the colour of the stimulus on the premise of ensuring correct reflection and then use the right finger to make a response. Stimulus words last for 1000 ms or disappear after pressing the button, and then the next test will start after 500 ms.

**Table 2:** Primary outcome of the three groups

	HIIT (*n=80*)			MICT (*n=80*)			CON (*n=80*)		
	Pre	Post	Follow-up	Pre	Post	Follow-up	Pre	Post	Follow-up
**Stroop**									
Congruent accuracy (%)									
Incongruent accuracy (%)									
Mean reaction time (ms)									
Congruent RT (ms)									
Incongruent RT (ms)									
Interference cost (ms)									
**Digit span**									
Number in order (min)									
Number in reverse order (min)									
**Symbol search**									
Symbol searched total									

**Fig. 3:**
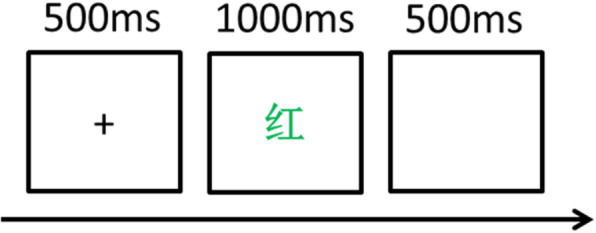
Stroop test

The whole task will be divided into two parts. The first part has a feedback, whereas the latter part has no feedback. The four stages will comprise 40 trials each in formal trials with a 30-s break in between. The three-colour words will be randomly presented to the participants for 24 times, and the symbols of red, green and blue are presented for 16 times. Each subject will receive a total of 120 tests. Response time and accuracy will be recorded at each stage. These two indicators represent an individual’s inhibition control ability (Fig. 3).

#### Wechsler Adult Intelligence Scale-III

The Wechsler Adult Intelligence Scale is the most commonly used test for assessing intelligence in various settings, including clinical practice [92]. Two tests from the Wechsler Adult Intelligence Scale-III [78], namely, digit span and symbol search, will be used to evaluate the following cognitive aspects: attention, working memory, processing speed, executive functions and cognitive flexibility.

##### Digit span

Digit span is a subtest in the Wechsler Adult Intelligence Scale-III that constitutes attention and working memory measures. The examiner pronounces a list of digits at a rate of approximately one digit per second, and the patients are required to repeat the list in the same order immediately. If they succeed, a list that is one digit longer is presented; if they fail, a second list of the same length is presented. If participants are successful on the second list, a list one digit longer is given as before. However, if they also fail on the second list, the test is ended. The length of the digit sequences increases gradually from a sequence of three numbers (e.g. 5, 8, 2) to a sequence with a maximum of nine items (e.g. 7, 1, 3, 9, 4, 2, 5, 6, 8). The span is established as the length of the most extended list recalled correctly.

##### Symbol search

This test evaluates attention, speed of mental processing, short-term visual memory, visual-motor coordination, cognitive flexibility, visual discrimination, psychomotor speed and speed of mental operation. It consists of 60 items. The task is to identify whether the symbols displayed in the target group (left) are present in the hunt group (right) within a specific time limit (120 s).

### Secondary outcome assessment

#### Cognitive function test

CogState battery (CSB), a computerised assessment tool, will be used to examine the different aspects of cognitive function. CSB has been applied to the substance-addicted population [17] with good reliability and validity. The following eight aspects of cognitive function will be examined using this tool: speech learning and memory (International Shopping List Task), processing speed (Detection Task), attention and vigilance (Identification Task), visual learning and memory (One Card Learning Task), working memory (Two Back Task), spatial working memory (Continuous Paired Association Learning Task), problem solving and wrong supervision (Groton Maze Learning Task) and social cognition (Social Emotional Cognition Task). All the tests will be conducted in a quiet room. The questions will be presented to the participants in a fixed order by a computer to reduce distractions, and each test will be scored automatically.

#### Craving Automated Scale for Substances (CAS-S)

The Chinese version of CAS-S will be used to assess the craving level and behaviours of the participants. The scale is a self-rating scale with 15 items. Each item has six points: 0 = never, 1 = hardly, 2 = occasionally, 3 = frequently, 4 = very frequently, and 5 = always. The use of addictive substances will be asked at the end of the questionnaire. This scale has been proven reliable and valid for the measurement of drug-craving, drug-seeking and drug use behaviours [77].

#### Mental health characteristics

Mental health characteristics will be collected by the Symptom Check List 90 (SCL-90), which is a psychosomatic screening scale compiled by Derogatis, Lipman, & Covi [24] that is widely used worldwide [3]. SCL-90 measures 10 kinds of mental symptoms, including somatisation, depression, anxiety, fear, compulsive symptoms and other factors. Each measurement item adopts a five-level grading system: (1) not at all, (2) minimal, (3) moderate, (4) severe and (5) extreme. The checklist will be used to assess how you actually feel ‘now’ or ‘in the last week’.

### Statistical analysis

SPSS 25.0 will be used for statistical analysis. The descriptive outcomes will be displayed as mean ± standard deviation. The *F*-test will be applied to test the continuous variables, and the chi-square test will be used to test the classification variables. For the Stroop test, reaction time (congruent and incongruent) and accuracy (congruent and incongruent) will be analysed using 2 (time points: pre-test vs. post-test) × 3 (groups: HIIT vs. MICT vs. control) repeated-measures ANOVA. Other data will also be analysed using repeated-measures ANOVA. All post hoc tests will be corrected by Bonferroni for multiple comparisons. Intention-to-treat analysis will be used to process the missing data. The linear mixed model method will be applied to analyse all continuous variables with random missing values. The magnitude of the difference between fitness levels will be assessed by Hedges’ *g* [28], and the magnitude will be classified as small (0.2 < ES < 0.5), moderate (0.5 < ES < 0.8) or large (ES > 0.8). The significance level will be set at *p* < 0.05 for all analyses.

### Data collection, management and monitoring

Study data will be collected during each assessment and electronically recorded in the laptop of the research team. Both electric and print-out data will be locked to prevent changes once recorded. Only the principal investigator and research team members can obtain permission to access the study data. After publication, all data will be stored in a removable storage device and locked in a drawer. Data of this trial will be monitored by the leader of the research team and is independent from the sponsor without competing interests.

### Protocol amendments

The sponsor, patients and the public will not be involved in the design, recruitment or conduct of this study. The opinion (including the acceptability of the intervention and the choice of assessment tools) of patients will be collected through an interview as this part is important in the process evaluation if the protocol needs amendments.

## Discussion

The conjunction of exercise and medicine therapy is a vital means of drug withdrawal management [5, 59]. MA dependents demonstrate poor functional performance in several specific domains, including comprehension and planning, financial transactions and communication skills, and often exhibit poor cognitive control, decision-making and social adjustment [23, 44, 52]. However, this performance can be ameliorated by exercise training. Preliminary studies suggest that HIIT may be superior to low-intensity exercise in improving and maintaining executive function [22, 51, 84]. A longer training time may have a stronger effect on cognitive function [82]. One research proved that obese patients’ cognitive functions, such as short-term memory, verbal memory, processing speed and attention, remarkably improved after 6 months of HIIT intervention [27]. Another study even showed that 2 weeks of HIIT has a mixed effect on adolescents’ cognition [82]. HIIT can also improve the inhibition of mentally ill youth by improving the response efficiency in the Stroop test control [57]. Moreover, studies reported that HIIT is more effective than continuous aerobic exercise in improving control task performance as a result of longer sustained benefits and increased neural efficiency for healthy adults [51, 84].

The possible effect of HIIT on the improvement of cognitive function can be explained by the following mechanisms. Firstly, exercise may increase cerebral blood flow. The cardiovascular hypothesis suggests that improvements in cardiovascular function (cardiac output, oxygen transport and metabolism) can improve neurotransmitter function and brain health [29]. According to this hypothesis, a higher cardiac output indicates higher cerebral blood flow, which means that HIIT is more adaptable to the cardiovascular system and has a positive effect on cognitive ability [65]. HIIT induces a greater increase in cardiac output than MICT [43]. Thus, this difference may be the reason why HIIT is better than MICT in improving cognitive function. The second mechanism is related to the increased level of brain-derived neurotrophic factor (BDNF) in the brain. Acute aerobic exercise increases the levels of BDNF, which is an important component of the brain’s neuroplasticity [34, 53]. Physical activity can improve the circulation level of BDNF, which has beneficial neurotrophic, neuroprotective and cognitive properties [88]. The impact of physical exercise on BDNF has been well summarised by some published reviews. Current evidence supports that physical exercise may change the concentration of BDNF in the brain; acute and chronic aerobic exercises increase peripheral BDNF concentration [21, 53, 64]. Specifically, BDNF level increases after short-term moderate-intensity exercise [33]. Mindfulness meditation and physical and mental exercises, including yoga and TC, can increase circulating BDNF in healthy and sick individuals [16, 94]. In addition, a new study shows [1] that a 3-week HIIT can bring about more BDNF expression and delivery in the brain and plasma. A similar study also demonstrated that HIIT can attenuate hippocampal oxidative damage by increasing BDNF concentration [37].

Exercise training interventions can increase the basal BDNF of healthy adults [1, 25], and BDNF has long been implicated in cognitive benefits [56]. Training-induced changes in the hippocampal and peripheral levels of BDNF also mediate cognitive improvement in animal models [86] and human models [56]. Notably, a recent study examined the relationship between BDNF and cognition and found that the level of BDNF in the brain is related to the cognitive area [46]. Another research on the relationship amongst BDNF levels, physical activity and cognition also believes that high-intensity short-term activities may effectively promote BDNF response and extend brain health from a practical perspective [88]. Through these findings, we speculate that the improvement of cognitive function in patients with cognitive deficits may be caused by an exercise-induced increase in BDNF levels, and high-intensity aerobic exercise is able to increase the level of this substance in the brain.

Whether HIIT is a suitable exercise for MA dependents is controversial. Some researchers believe that interval training may be too arduous and may give participants a feeling of incompetence, failure and inferiority, which may reduce the participants’ motivation to participate in physical exercise [42]. However, some studies conducted on adults with mental illness have shown that HIIT is as feasible as continuous aerobic exercise in terms of withdrawal rate, self-determined exercise motivation and emotional response after exercise [10, 39]. HIIT consists of customised exercise prescriptions that are feasible in most exercise environments [76]. Most exercise interventions for substance abuse have low or moderate intensity, and these interventions may exert a positive effect on cognitive function. However, the effectiveness brought by high-intensity exercise is considered better than that of moderate-intensity exercise [19] apart from the potential risks brought by HIIT [74]. Thus, the ultimate results of this trial may find HIIT has a better effect on the cognitive functions and drug craving level of individuals with MA dependence than traditional interventions. The results may provide new evidences for the use of HIIT amongst illicit drug abusers.

This study has several limitations. Firstly, a blinded intervention is difficult to achieve because the exercise that will serve as the intervention is widely open to the administrator and participants. Secondly, the subjects in this trial are restricted to MA dependents, therefore, the effect of HIIT on other participants that use a different substance other than MA remains unknown. Thirdly, the functions of cardiorespiratory and other organs of the body need to be mobilised during the HIIT intervention. Hence, future research should concentrate on how to reduce the risk of HIIT intervention on healthy and diseased subjects.

## Trial status

The trial was initiated as planned in April 2020. The protocol version number is 2020-4 (date: May 31, 2020). This trial was originally scheduled to be completed in July 2021. However, our trial has been delayed to a certain extent because of the outbreak and persistence of COVID-19 in 2020. The recruitment is currently ongoing.

## Data Availability

The data from this study will be kept by the research team when the study is finished. All principal investigators will be given access to the cleaned data sets. After the publication of this study, the data will be available to researchers or the public when requested.

## Abbreviations

MA: Methamphetamine
TC: Tai chi
HIIT: High-intensity interval training
MICT: Moderate-intensity continuous training
SMDRC: Shanghai Mandatory Detoxification and Rehabilitation Center
HR: Heart rate
WISC-III: Wechsler Adult Intelligence Scale-III
CSB: CogState battery
CAS-S: Craving Automated Scale for Substances
PFC: Prefrontal cortex
BDNF: Brain-derived neurotrophic factor

## References

[1] Abbasian S, Asghar Ravasi A. The effect of antecedent-conditioning high-intensity interval training on BDNF regulation through PGC-1α pathway following cerebral ischemia. Brain Res. 2020;1729:146618. 10.1016/j.brainres.2019.146618. 10.1016/j.brainres.2019.146618 31866362

[2] Abrantes AM, Blevins CE. Exercise in the context of substance use treatment: key issues and future directions. Curr Opin Psychol. 2019;30:103–8. 10.1016/j.copsyc.2019.04.001. 10.1016/j.copsyc.2019.04.001 31079012

[3] Achenbach TM, Ivanova MY, Rescorla LA, Turner LV, Althoff RR. Internalizing/externalizing problems: review and recommendations for clinical and research applications. J Am Acad Child Adolesc Psychiatr. 2016:647–56. 10.1016/j.jaac.2016.05.012. 10.1016/j.jaac.2016.05.012 27453078

[4] Alizadehgoradel J, Nejati V, Sadeghi Movahed F, Imani S, Taherifard M, Mosayebi-Samani M, et al. Repeated stimulation of the dorsolateral-prefrontal cortex improves executive dysfunctions and craving in drug addiction: a randomized, double-blind, parallel-group study. Brain Stimul. 2020;13(3):582–93. 10.1016/j.brs.2019.12.028. 10.1016/j.brs.2019.12.028 32289681

[5] Ashdown-Franks G, Firth J, Carney R, Carvalho AF, Hallgren M, Koyanagi A, et al. Exercise as medicine for mental and substance use disorders: a meta-review of the benefits for neuropsychiatric and cognitive outcomes. Sports Med. 2020;50(1):151–70. 10.1007/s40279-019-01187-6. 10.1007/s40279-019-01187-6 31541410

[6] Bahdur K, Gilchrist R, Park G, Nina L, Pruna R. Effect of HIIT on cognitive and physical performance. Apunts Med l’Esport. 2019;54(204):113–7. 10.1016/j.apunts.2019.07.001.

[7] Baicy K, London ED. Corticolimbic dysregulation and chronic methamphetamine abuse. Addiction. 2007;102(Suppl 1):5–15. 10.1111/j.1360-0443.2006.01777.x. 10.1111/j.1360-0443.2006.01777.x 17493049

[8] Balady GJ, Arena R, Sietsema K, Myers J, Coke L, Fletcher GF, et al. Clinician’s guide to cardiopulmonary exercise testing in adults: a scientific statement from the American Heart Association. Circulation. 2010;122(2):191–225. 10.1161/CIR.0b013e3181e52e69. 10.1161/CIR.0b013e3181e52e69 20585013

[9] Baler RD, Volkow ND. Drug addiction: the neurobiology of disrupted self-control. Trends Mol Med. 2006;12(12):559–66. 10.1016/j.molmed.2006.10.005. 10.1016/j.molmed.2006.10.005 17070107

[10] Bartlett JD, Close GL, MacLaren DP, Gregson W, Drust B, Morton JP. High-intensity interval running is perceived to be more enjoyable than moderate-intensity continuous exercise: implications for exercise adherence. J Sports Sci. 2011;29(6):547–53. 10.1080/02640414.2010.545427. 10.1080/02640414.2010.545427 21360405

[11] Bechara A, Tranel D, Damasio H. Characterization of the decision-making deficit of patients with ventromedial prefrontal cortex lesions. Brain. 2000;123(Pt 11):2189–202. 10.1093/brain/123.11.2189. 10.1093/brain/123.11.2189 11050020

[12] Bernheim A, See RE, Reichel CM. Chronic methamphetamine self-administration disrupts cortical control of cognition. Neurosci Biobehav Rev. 2016;69:36–48. 10.1016/j.neubiorev.2016.07.020. 10.1016/j.neubiorev.2016.07.020 PMC5030184 27450578

[13] Bock BC, Marcus BH, King TK, Borrelli B, Roberts MR. Exercise effects on withdrawal and mood among women attempting smoking cessation. Addict Behav. 1999;24(3):399–410. 10.1016/s0306-4603(98)00088-4. 10.1016/s0306-4603(98)00088-4 10400278

[14] Borg GA. Psychophysical bases of perceived exertion. Med Sci Sports Exerc. 1982;14(5):377–81. 10.1249/00005768-198205000-00012. 7154893

[15] Buchheit M, Laursen PB. High-intensity interval training, solutions to the programming puzzle: part I: cardiopulmonary emphasis. Sports Med. 2013;43(5):313–38. 10.1007/s40279-013-0029-x. 10.1007/s40279-013-0029-x 23539308

[16] Cahn BR, Goodman MS, Peterson CT, Maturi R, Mills PJ. Yoga, Meditation and mind-body health: increased BDNF, cortisol awakening response, and altered inflammatory marker expression after a 3-month yoga and meditation retreat. Front Hum Neurosci. 2017;11:315. 10.3389/fnhum.2017.00315. 10.3389/fnhum.2017.00315 PMC5483482 28694775

[17] Caroline D, Rae Joanne E, Davidson B. Ethanol, not detectably metabolized in brain, significantly reduces brain metabolism, probably via action at specific GABA(A) receptors and has measureable metabolic effects at very low concentrations. J Neurochem. 2013;129(2):304–14. 10.1111/jnc.12634. 10.1111/jnc.12634 PMC3980046 24313287

[18] Chang YK, Labban JD, Gapin JI, Etnier JL. The effects of acute exercise on cognitive performance: a meta-analysis. Brain Res. 2012;1453:87–101. 10.1016/j.brainres.2012.02.068. 10.1016/j.brainres.2012.02.068 22480735

[19] Chen Y, Zhou Y, Wang J, Zhou C, Lu Y (2019). Effects of acute aerobic exercise on drug craving among methamphetamine abstainers and moderation effect of cognitive function. Chin J Drug Depend.

[20] Chunguang W, Ming Y, Yonghui L, Nan S. Identifying the features of attention bias in methamphetamine addicts: a study with words emotional Stroop task. Chin J Drug Depend. 2015;24(05):391–5.

[21] Coelho FG, Gobbi S, Andreatto CA, Corazza DI, Pedroso RV, Santos-Galduróz RF. Physical exercise modulates peripheral levels of brain-derived neurotrophic factor (BDNF): a systematic review of experimental studies in the elderly. Arch Gerontol Geriatr. 2013;56(1):10–5. 10.1016/j.archger.2012.06.003. 10.1016/j.archger.2012.06.003 22749404

[22] Cooper SL, Tomporowski PD. Acute effects of exercise on attentional bias in low and high anxious young adults. Ment Health Phys Act. 2017;12:62–72. 10.1016/j.mhpa.2017.02.002.

[23] Dean AC, Groman SM, Morales AM, London ED. An evaluation of the evidence that methamphetamine abuse causes cognitive decline in humans. Neuropsychopharmacol. 2013;38(2):259–74. 10.1038/npp.2012.179. 10.1038/npp.2012.179 PMC3527116 22948978

[24] Derogatis LR, Lipman RS, Covi L (1973). The SCL-90: an outpatient psychiatric rating scale. Psychopharmacol Bull.

[25] Dinoff A, Herrmann N, Swardfager W, Lanctôt KL. The effect of acute exercise on blood concentrations of brain-derived neurotrophic factor in healthy adults: a meta-analysis. Eur J Neurosci. 2017;46(1):1635–46 10.1111/ejn.13603. 10.1111/ejn.13603 28493624

[26] Dong G, Zhou H, Zhao X. Male Internet addicts show impaired executive control ability: evidence from a color-word Stroop task. Neurosci Lett. 2011;499(2):114–8. 10.1016/j.neulet.2011.05.047. 10.1016/j.neulet.2011.05.047 21645588

[27] Drigny J, Gremeaux V, Dupuy O, Gayda M, Bherer L, Juneau M, et al. Effect of interval training on cognitive functioning and cerebral oxygenation in obese patients: a pilot study. J Rehabil Med. 2014;46(10):1050–4. 10.2340/16501977-1905. 10.2340/16501977-1905 25297458

[28] Dupuy O, Lussier M, Fraser S, Bherer L, Audiffren M, Bosquet L. Effect of overreaching on cognitive performance and related cardiac autonomic control. Scand J Med Sci Sports. 2014;24(1):234–42. 10.1111/j.1600-0838.2012.01465.x. 10.1111/j.1600-0838.2012.01465.x 22537000

[29] Dustman RE, Emmerson RY, Ruhling RO, Shearer DE, Steinhaus LA, Johnson SC, et al. Age and fitness effects on EEG, ERPs, visual sensitivity, and cognition. Neurobiol Aging. 1990;11(3):193–200. 10.1016/0197-4580(90)90545-b. 10.1016/0197-4580(90)90545-b 2362652

[30] Etnier JL, Wideman L, Labban JD, Piepmeier AT, Pendleton DM, Dvorak KK, et al. The effects of acute exercise on memory and brain-derived neurotrophic factor (BDNF). J Sport Exerc Psychol. 2016;38(4):331–40. 10.1123/jsep.2015-0335. 10.1123/jsep.2015-0335 27385735

[31] Fehr T, Wiedenmann P, Herrmann M. Nicotine Stroop and addiction memory--an ERP study. Int J Psychophysiol. 2006;62(2):224–32. 10.1016/j.ijpsycho.2006.01.011. 10.1016/j.ijpsycho.2006.01.011 16492391

[32] Feil J, Sheppard D, Fitzgerald PB, Yücel M, Lubman DI, Bradshaw JL. Addiction, compulsive drug seeking, and the role of frontostriatal mechanisms in regulating inhibitory control. Neurosci Biobehav Rev. 2010;35(2):248–75. 10.1016/j.neubiorev.2010.03.001. 10.1016/j.neubiorev.2010.03.001 20223263

[33] Ferreira AF, Real CC, Rodrigues AC, Alves AS, Britto LR. Short-term, moderate exercise is capable of inducing structural, BDNF-independent hippocampal plasticity. Brain Res. 2011;1425:111–22. 10.1016/j.brainres.2011.10.004. 10.1016/j.brainres.2011.10.004 22035567

[34] Ferris LT, Williams JS, Shen CL. The effect of acute exercise on serum brain-derived neurotrophic factor levels and cognitive function. Med Sci Sports Exerc. 2007;39(4):728–34. 10.1249/mss.0b013e31802f04c7. 10.1249/mss.0b013e31802f04c7 17414812

[35] Field M, Christiansen P, Cole J, Goudie A. Delay discounting and the alcohol Stroop in heavy drinking adolescents. Addiction. 2007;102(4):579–86. 10.1111/j.1360-0443.2007.01743.x. 10.1111/j.1360-0443.2007.01743.x 17309540

[36] Fiorelli CM, Ciolac EG, Simieli L, Silva FA, Fernandes B, Christofoletti G, et al. Differential acute effect of high-intensity interval or continuous moderate exercise on cognition in individuals with Parkinson’s disease. J Phys Act Health. 2019;16(2):157–64. 10.1123/jpah.2018-0189. 10.1123/jpah.2018-0189 30626260

[37] Freitas DA, Rocha-Vieira E, Soares BA, Nonato LF, Fonseca SR, Martins JB, et al. High intensity interval training modulates hippocampal oxidative stress, BDNF and inflammatory mediators in rats. Physiol Behav. 2018;184:6–11. 10.1016/j.physbeh.2017.10.027. 10.1016/j.physbeh.2017.10.027 29111230

[38] Garavan H, Weierstall K. The neurobiology of reward and cognitive control systems and their role in incentivizing health behavior. Prev Med. 2012;55(Suppl):S17–23. 10.1016/j.ypmed.2012.05.018. 10.1016/j.ypmed.2012.05.018 22683229

[39] Gerber M, Minghetti A, Beck J, Zahner L, Donath L. Sprint interval training and continuous aerobic exercise training have similar effects on exercise motivation and affective responses to exercise in patients with major depressive disorders: a randomized controlled trial. Front Psychiatry. 2018;9:694. 10.3389/fpsyt.2018.00694. 10.3389/fpsyt.2018.00694 PMC6308196 30622487

[40] Gibala MJ, McGee SL. Metabolic adaptations to short-term high-intensity interval training: a little pain for a lot of gain? Exerc Sport Sci Rev. 2008;36(2):58–63. 10.1097/JES.0b013e318168ec1f. 10.1097/JES.0b013e318168ec1f 18362686

[41] Groman SM, James AS, Jentsch JD. Poor response inhibition: at the nexus between substance abuse and attention deficit/hyperactivity disorder. Neurosci Biobehav Rev. 2009;33(5):690–8. 10.1016/j.neubiorev.2008.08.008. 10.1016/j.neubiorev.2008.08.008 PMC2728075 18789354

[42] Hardcastle SJ, Ray H, Beale L, Hagger MS. Why sprint interval training is inappropriate for a largely sedentary population. Front Psychol. 2014;5:1505. 10.3389/fpsyg.2014.01505. 10.3389/fpsyg.2014.01505 PMC4274872 25566166

[43] Helgerud J, Høydal K, Wang E, Karlsen T, Berg P, Bjerkaas M, et al. Aerobic high-intensity intervals improve VO2max more than moderate training. Med Sci Sports Exerc. 2007;39(4):665–71. 10.1249/mss.0b013e3180304570. 10.1249/mss.0b013e3180304570 17414804

[44] Henry BL, Minassian A, Perry W. Effect of methamphetamine dependence on everyday functional ability. Addict Behav. 2010;35(6):593–8. 10.1016/j.addbeh.2010.01.013. 10.1016/j.addbeh.2010.01.013 PMC2839012 20167435

[45] Hester R, Dixon V, Garavan H. A consistent attentional bias for drug-related material in active cocaine users across word and picture versions of the emotional Stroop task. Drug Alcohol Depend. 2006;81(3):251–7. 10.1016/j.drugalcdep.2005.07.002. 10.1016/j.drugalcdep.2005.07.002 16095852

[46] Hori H, Yoshimura R, Katsuki A, Atake K, Igata R, Konishi Y, et al. Blood biomarkers predict the cognitive effects of aripiprazole in patients with acute schizophrenia. Int J Mol Sci. 2017;18(3). 10.3390/ijms18030568. 10.3390/ijms18030568 PMC5372584 28272307

[47] Huang H, Wang KK, Zhou N, Zhou L, Le ping LI, Ding LS, Zhou F. Influence of various intensities of exercise training on plasma β-endorphin peptide in rats addictive to heroin. Chin J Phys Med Rehabil. 2004;(09):11–13.

[48] Iudicello JE, Woods SP, Vigil O, Scott JC, Cherner M, Heaton RK, et al. Longer term improvement in neurocognitive functioning and affective distress among methamphetamine users who achieve stable abstinence. J Clin Exp Neuropsychol. 2010;32(7):704–18. 10.1080/13803390903512637. 10.1080/13803390903512637 PMC2911490 20198527

[49] Jingjing G (2016). Effect of tai chi rehabilitation exercise on the rehabilitation of female drug addicts in compulsory isolation. Chin J Sports Med.

[50] Kalechstein AD, Newton TF, Green M. Methamphetamine dependence is associated with neurocognitive impairment in the initial phases of abstinence. J Neuropsychiatry Clin Neurosci. 2003;15(2):215–20. 10.1176/jnp.15.2.215. 10.1176/jnp.15.2.215 12724464

[51] Kao SC, Westfall DR, Soneson J, Gurd B, Hillman CH. Comparison of the acute effects of high-intensity interval training and continuous aerobic walking on inhibitory control. Psychophysiology. 2017;54(9):1335–45. 10.1111/psyp.12889. 10.1111/psyp.12889 28480961

[52] Kim YT, Kwon DH, Chang Y. Impairments of facial emotion recognition and theory of mind in methamphetamine abusers. Psychiatry Res. 2011;186(1):80–4.10.1016/j.psychres.2010.06.027. 10.1016/j.psychres.2010.06.027 20643485

[53] Knaepen K, Goekint M, Heyman EM, Meeusen R. Neuroplasticity - exercise-induced response of peripheral brain-derived neurotrophic factor: a systematic review of experimental studies in human subjects. Sports Med. 2010;40(9):765–801. 10.2165/11534530-000000000-00000. 10.2165/11534530-000000000-00000 20726622

[54] Laurin D, Verreault R, Lindsay J, MacPherson K, Rockwood K. Physical activity and risk of cognitive impairment and dementia in elderly persons. Arch Neurol. 2001;58(3):498–504. 10.1001/archneur.58.3.498. 10.1001/archneur.58.3.498 11255456

[55] Le X, Yuan XF, Yan KJ, Cui SJ, Ya-Qiong LI, Zhang GF, et al. Decision-making impairment caused by chronic methamphetamine use. J Clin Psychiatry. 2016;026(002):112–4.

[56] Leckie RL, Oberlin LE, Voss MW, Prakash RS, Szabo-Reed A, Chaddock-Heyman L, et al. BDNF mediates improvements in executive function following a 1-year exercise intervention. Front Hum Neurosci. 2014;8:985. 10.3389/fnhum.2014.00985. 10.3389/fnhum.2014.00985 PMC4263078 25566019

[57] Lee JS, Boafo A, Greenham S, Longmuir PE. The effect of high-intensity interval training on inhibitory control in adolescents hospitalized for a mental illness. Ment Health Phys Act. 2019;17:100298. 10.1016/j.mhpa.2019.100298.

[58] Li G, Li N, Zheng W, Wang Q (2011). Comparative study on the psychological and behavioral characteristics between “new drug” abusers and “traditional drug” abusers. Chin J Drug Depend.

[59] Linke SE, Ussher M. Exercise-based treatments for substance use disorders: evidence, theory, and practicality. Am J Drug Alcohol Abuse. 2015;41(1):7–15. 10.3109/00952990.2014.976708. 10.3109/00952990.2014.976708 PMC4831948 25397661

[60] Loprinzi PD, Frith E, Edwards MK, Sng E, Ashpole N. The effects of exercise on memory function among young to middle-aged adults: systematic review and recommendations for future research. Am J Health Promot. 2018;32(3):691–704. 10.1177/0890117117737409. 10.1177/0890117117737409 29108442

[61] Lusher J, Chandler C, Ball D. Alcohol dependence and the alcohol Stroop paradigm: evidence and issues. Drug Alcohol Depend. 2004;75(3):225–31. 10.1016/j.drugalcdep.2004.03.004. 10.1016/j.drugalcdep.2004.03.004 15283943

[62] Lynch WJ, Peterson AB, Sanchez V, Abel J, Smith MA. Exercise as a novel treatment for drug addiction: a neurobiological and stage-dependent hypothesis. Neurosci Biobehav Rev. 2013;37(8):1622–44. 10.1016/j.neubiorev.2013.06.011. 10.1016/j.neubiorev.2013.06.011 PMC3788047 23806439

[63] MacLeod CM. Half a century of research on the Stroop effect: an integrative review. Psychol Bull. 1991;109(2):163–203. 10.1037/0033-2909.109.2.163. 10.1037/0033-2909.109.2.163 2034749

[64] Mang CS, Campbell KL, Ross CJ, Boyd LA. Promoting neuroplasticity for motor rehabilitation after stroke: considering the effects of aerobic exercise and genetic variation on brain-derived neurotrophic factor. Phys Ther. 2013;93(12):1707–16. 10.2522/ptj.20130053. 10.2522/ptj.20130053 PMC3870490 23907078

[65] Mekari S, Earle M, Martins R, Drisdelle S, Killen M, Bouffard-Levasseur V, et al. Effect of high intensity interval training compared to continuous training on cognitive performance in young healthy adults: a pilot study. Brain Sci. 2020;10(2):81. 10.3390/brainsci10020081. 10.3390/brainsci10020081 PMC7071608 32033006

[66] Mingzhen Z. Effect of tai chi rehabilitation exercise on heart rate variability and related indexes of female methamphetamine dependent indualvidus. Shanghai University of Sport. 2018;01:43.

[67] Mitchell MR, Potenza MN. Stroop, cocaine dependence, and intrinsic connectivity. Neurosci Cocaine. 2017;331:339. 10.1016/B978-0-12-803750-8.00034-8.

[68] Mizoguchi H, Katahira K, Inutsuka A, Fukumoto K, Nakamura A, Wang T, et al. Insular neural system controls decision-making in healthy and methamphetamine-treated rats. Proc Natl Acad Sci U S A. 2015;112(29):E3930–9. 10.1073/pnas.1418014112. 10.1073/pnas.1418014112 PMC4517258 26150496

[69] Mizoguchi H, Wang T, Kusaba M, Fukumoto K, Yamada K. Nicotine and varenicline ameliorate changes in reward-based choice strategy and altered decision-making in methamphetamine-treated rats. Behav Brain Res. 2019;359:935–41. 10.1016/j.bbr.2018.06.016. 10.1016/j.bbr.2018.06.016 29935276

[70] Mizoguchi H, Yamada K. Methamphetamine use causes cognitive impairment and altered decision-making. Neurochem Int. 2019;124:106–13. 10.1016/j.neuint.2018.12.019. 10.1016/j.neuint.2018.12.019 30611760

[71] Office of China National Narcotics Control Commission Publication.(2020). China’s drug situation report, 2020. from http://www.gov.cn/xinwen/2020-06/28/content_5522443.htm.

[72] Portugal ACA, Afonso AS Jr, Caldas AL, Maturana W, Mocaiber I, Machado-Pinheiro W. Inhibitory mechanisms involved in Stroop-matching and stop-signal tasks and the role of impulsivity. Acta Psychol (Amst). 2018;191:234–43. 10.1016/j.actpsy.2018.10.003. 10.1016/j.actpsy.2018.10.003 30343096

[73] Potvin S, Pelletier J, Grot S, Hébert C, Barr AM, Lecomte T. Cognitive deficits in individuals with methamphetamine use disorder: a meta-analysis. Addict Behav. 2018;80:154–60. 10.1016/j.addbeh.2018.01.021. 10.1016/j.addbeh.2018.01.021 29407687

[74] Quindry JC, Franklin BA, Chapman M, Humphrey R, Mathis S. Benefits and risks of high-intensity interval training in patients with coronary artery disease. Am J Cardiol. 2019;123(8):1370–7. 10.1016/j.amjcard.2019.01.008. 10.1016/j.amjcard.2019.01.008 30732854

[75] Romain AJ, Fankam C, Karelis AD, Letendre E, Mikolajczak G, Stip E, et al. Effects of high intensity interval training among overweight individuals with psychotic disorders: a randomized controlled trial. Schizophr Res. 2019;210:278–86. 10.1016/j.schres.2018.12.021. 10.1016/j.schres.2018.12.021 30595443

[76] Ross LM, Porter RR, Durstine JL. High-intensity interval training (HIIT) for patients with chronic diseases. J Sport Health Sci. 2016;5(2):139–44. 10.1016/j.jshs.2016.04.005. 10.1016/j.jshs.2016.04.005 PMC6188712 30356536

[77] Ruiting Y, Pengfei W, Wenjun T, Zhiling Z, Hong Z (2019). Reliability and validity of the Craving Automated Scale for substances in Chinese drug addicts. Chin J Clin Psychol.

[78] Ryan JJ, Lopez SJ. Wechsler Adult Intelligence Scale-III. In: Dorfman W.I., Hersen M. (eds) Understanding Psychological Assessment. Perspectives on Individual Differences. Boston: Springer; 2001. 10.1007/978-1-4615-1185-4_2.

[79] Salo R, Nordahl TE, Possin K, Leamon M, Gibson DR, Galloway GP, et al. Preliminary evidence of reduced cognitive inhibition in methamphetamine-dependent individuals. Psychiatry Res. 2002;111(1):65–74. 10.1016/s0165-1781(02)00111-7. 10.1016/s0165-1781(02)00111-7 12140121

[80] Scott JC, Woods SP, Matt GE, Meyer RA, Heaton RK, Atkinson JH, et al. Neurocognitive effects of methamphetamine: a critical review and meta-analysis. Neuropsychol Rev. 2007;17(3):275–97. 10.1007/s11065-007-9031-0. 10.1007/s11065-007-9031-0 17694436

[81] Shumei Z. Effects of exercise on psychological function of detoxification women with heroin dependence. Tianjin Medical University. 2013;01:95.

[82] Stenman M, Pesola AJ, Laukkanen A, Haapala EA. Effects of two-week high intensity interval training on cognition in adolescents: randomized controlled pilot study. Hum Mov. 2017;18(2). 10.1515/humo-2017-0019.

[83] Taylor AH, Ussher MH, Faulkner G. The acute effects of exercise on cigarette cravings, withdrawal symptoms, affect and smoking behaviour: a systematic review. Addiction. 2007;102(4):534–43. 10.1111/j.1360-0443.2006.01739.x. 10.1111/j.1360-0443.2006.01739.x 17286639

[84] Tsukamoto H, Suga T, Takenaka S, Tanaka D, Takeuchi T, Hamaoka T, et al. Greater impact of acute high-intensity interval exercise on post-exercise executive function compared to moderate-intensity continuous exercise. Physiol Behav. 2016;155:224–30. 10.1016/j.physbeh.2015.12.021. 10.1016/j.physbeh.2015.12.021 26723268

[85] United Nations Office on Drugs and Crime, U (2017). World drug report 2017.

[86] Vaynman S, Ying Z, Gomez-Pinilla F. Hippocampal BDNF mediates the efficacy of exercise on synaptic plasticity and cognition. Eur J Neurosci. 2004;20(10):2580–90. 10.1111/j.1460-9568.2004.03720.x. 10.1111/j.1460-9568.2004.03720.x 15548201

[87] Volkow ND, Li TK. Drug addiction: the neurobiology of behaviour gone awry. Nat Rev Neurosci. 2004;5(12):963–70. 10.1038/nrn1539. 10.1038/nrn1539 15550951

[88] Walsh EI, Smith L, Northey J, Rattray B, Cherbuin N. Towards an understanding of the physical activity-BDNF-cognition triumvirate: a review of associations and dosage. Ageing Res Rev. 2020;60:101044. 10.1016/j.arr.2020.101044. 10.1016/j.arr.2020.101044 32171785

[89] Wang D, Wang Y, Wang Y, Li R, Zhou C. Impact of physical exercise on substance use disorders: a meta-analysis. PLoS One. 2014;9(10):e110728. 10.1371/journal.pone.0110728. 10.1371/journal.pone.0110728 PMC4199732 25330437

[90] Wang D, Zhu T, Zhou C, Chang YK. Aerobic exercise training ameliorates craving and inhibitory control in methamphetamine dependencies: a randomized controlled trial and event-related potential study. Psychol Sport Exerc. 2017;30:82–90. 10.1016/j.psychsport.2017.02.001.

[91] Wang J, Chen T. Inhibitory control and higher cognitive functions. Adv Psychol Sci. 2013;20:1768–78. 10.3724/SP.J.1042.2012.01768.

[92] Wechsler D. Wechsler Adult Intelligence Scale–fourth edition (WAIS–IV). WAIS-IV administration and scoring manual. San Antonio, TX: Pearson. 2008.

[93] Weinstock J, Farney MR, Elrod NM, Henderson CE, Weiss EP. Exercise as an adjunctive treatment for substance use disorders: rationale and intervention description. J Subst Abuse Treat. 2017;72:40–7. 10.1016/j.jsat.2016.09.002. 10.1016/j.jsat.2016.09.002 PMC5289308 27666958

[94] You T, Ogawa EF. Effects of meditation and mind-body exercise on brain-derived neurotrophic factor: a literature review of human experimental studies. Sports Med Health Sci. 2020. 10.1016/j.smhs.2020.03.001. 10.1016/j.smhs.2020.03.001 PMC9219319 35783336

[95] Zhao H, Huang X, He Q. The cognitive impairment associated with substance addiction and its neural basis. Chin Sci Bull. 2016;61(34):3672–83. 10.1360/N972016-00533.

[96] Zhong N, Jiang H, Du J, Zhao Y, Sun H, Xu D, et al. The cognitive impairments and psychological wellbeing of methamphetamine dependent patients compared with health controls. Prog Neuropsychopharmacol Biol Psychiatry. 2016;69:31–7. 10.1016/j.pnpbp.2016.04.005. 10.1016/j.pnpbp.2016.04.005 27085943

[97] Dong Z, Ding X, Dai G, Jingjing G. The physical and mental effects of tai chi for synthetic drug abusers. Chinese J Drug Dependence. 2016;25(03):284–90.

[98] Zou H, Guo R, Zheng LI. Survey on knowledge, attitude and drug abuse intention among methamphetamine abusers. Chin J Drug Depend. 2012. 10.1007/s11783-011-0280-z.

